# A Case Series of Gastrointestinal Tuberculosis in Renal Transplant Patients

**DOI:** 10.1155/2013/213273

**Published:** 2013-02-24

**Authors:** Pedro Azevedo, Cristina Freitas, Hugo Silva, Pedro Aguiar, Pedro Farrajota, Manuela Almeida, Sofia Pedroso, La Salete Martins, Leonídio Dias, José Ramón Vizcaíno, António Castro Henriques, António Cabrita

**Affiliations:** ^1^Department of Nephrology, Centro Hospitalar do Porto, Santo António Hospital, 4099-001 Porto, Portugal; ^2^Department of Pathology, Centro Hospitalar do Porto, Santo António Hospital, 4099-001 Porto, Portugal

## Abstract

Tuberculosis is a disease relatively frequent in renal transplant patients, presenting a wide variety of clinical manifestations, often involving various organs and potentially fatal. Gastrointestinal tuberculosis, although rare in the general population, is about 50 times more frequent in renal transplant patients. Intestinal tuberculosis has a very difficult investigational approach, requiring a high clinical suspicion for its diagnosis. Therapeutic options may be a problem in the context of an immunosuppressed patient, requiring adjustment of maintenance therapy. The authors report two cases of isolated gastro-intestinal tuberculosis in renal transplant recipients that illustrates the difficulty of making this diagnosis and a brief review of the literature on its clinical presentation, diagnosis, and therapeutic approach.

## 1. Introduction

Mycobacterium tuberculosis (MT) is a common infectious agent, particularly in developing countries, with a reported incidence of 18.9 cases/100.000 inhabitants/year in general population [1–3]. The prevalence of tuberculosis (TB) in Portugal is high (34 cases per 100.000 inhabitants/year), corresponding to three times the average in Western Europe [4].

In transplanted patients the incidence of this opportunistic agent is even more frequent, with 512 cases/100.000 inhabitants/year and it is often linked to adverse outcomes [1–3].

In transplant recipients, MT infection can be due to primary infection, reactivation of latent TB foci favored by immunosuppression (IS), or, in a lesser extent (4%), it can be transmitted by the allograft [3, 5, 6].

In most cases, the disease involves the lungs. However, unlike general population, in renal transplant (RT) patients, extrapulmonar (occurring in 15%) and disseminated diseases (33–49%) are very frequent [1–3, 7]. In these patients atypical presentation is the rule and it requires a high clinical suspicion for its diagnosis [8]. Therapeutic options may be a problem in the context of an immunosuppressed patient, requiring frequent adjustment of maintenance therapy.

Delayed diagnosis of TB and drug interactions may contribute to extremely high mortality in RT recipients [7]. 

The authors report two cases of isolated gastrointestinal (GI) TB in RT recipients that illustrates the difficulty of its diagnosis and do a brief review of the literature on this topic.

## 2. Case 1

 A 53-year-old man with end-stage renal failure (ESRD) of unknown etiology was on hemodialysis (HD) since 1999. He underwent a first RT in 2000, with cyclosporine (CyA), mycophenolate mofetil (MMF), and prednisolone as immunosuppressive therapy. Renal graft was lost after 3 days, due to renal artery thrombosis. Second deceased kidney transplantation was performed in 2007. Both were from cytomegalovirus (CMV) positive donors and the recipient was CMV positive as well. Initial immunosuppressive therapy was daclizumab, prednisolone, MMF, and tacrolimus (TAC). In the immediate posttransplant period, there were no surgical or infectious complications, delayed graft function, or acute rejection episodes. Five months after RT, the patient had a CMV infection treated with ganciclovir with success.

Two years later, obstructive acute kidney injury was diagnosed, requiring a urological approach. Graft function stabilized to a serum creatinine (Cr) 2.3 mg/dL. 

**Figure 1:**
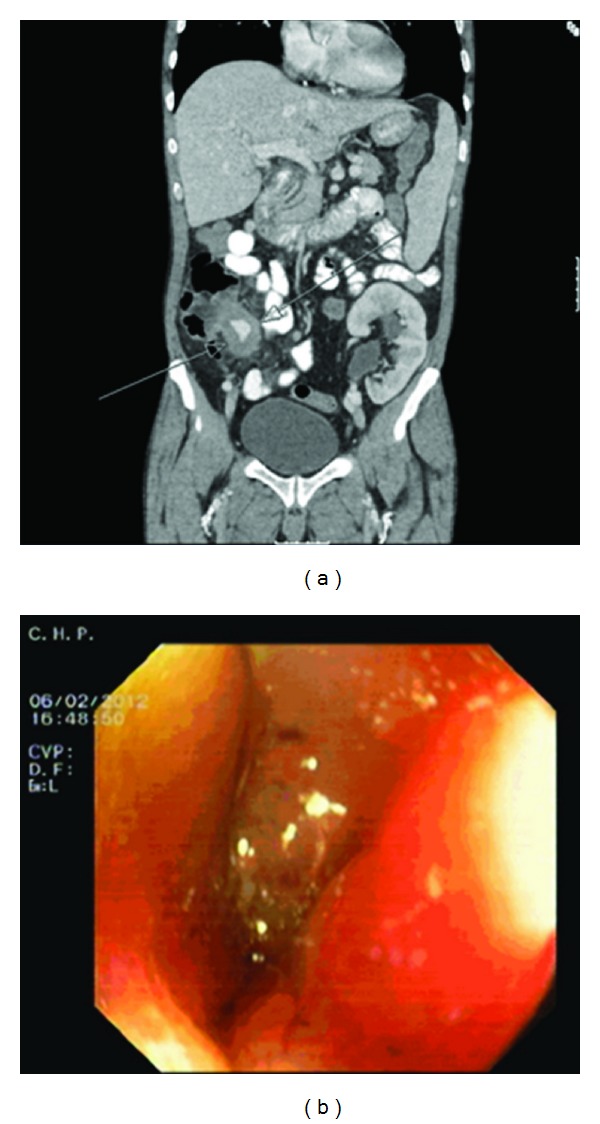
(a) Abdominal CT showing terminal ileitis. (b) Colonoscopy showing congestion and hyperemia of ileocecal valve.

Five years after RT, in February 2012, he was admitted with mild fever, profuse night sweating, and weight loss of 10% of his body weight, with three months of evolution. In the week prior to admission, he complained of pain in the right iliac fossa. The patient had no diarrhea, urinary symptoms, graft pain or other complaints. He had no recent travel history or known TB exposure. Analytical study revealed an elevated C-reactive protein (CRP = 70 mg/L), no anemia or leukocytosis, acute graft dysfunction, or other abnormalities. Serological study was negative, including viral hepatitis and HIV. Tuberculin skin testing was negative. Chest and abdominal X-ray and abdominal ultrasonography showed no abnormalities. Abdominal tomography (CT) was performed and showed terminal ileitis (Figure 1(a)). Colonoscopy revealed congestion and hyperemia of the ileocecal valve (ICV) with two small erosions (Figure 1(b)). ICV biopsy showed large bowel mucosa with irregularities of the surface epithelium and focal erosions, edema and congestion of the corium with mild to moderate inflammatory infiltrate. 

No viral inclusions, granulomas, or other morphological changes were identified, and Ziehl-Neelsen was negative. Polymerase chain reaction (PCR) for the diagnosis of TB on the biopsy tissue was positive.

Antituberculosis (anti-TB) therapy was started with rifampicin, isoniazid, pyrazinamide, and ethambutol, with clinical improvement. There was a need to increase by 7 times the dose of TAC, after the introduction of Rifampicin, without rejection episodes.

Nine months later, the patient is asymptomatic and the examination of the small bowel transit and colonoscopy showed no abnormalities and no evidence of reinfection.

## 3. Case 2

 A 53-year-old woman, inactive carrier of hepatitis B and ESRD secondary to membranoproliferative glomerulonephritis, was on HD since 1984. She underwent a deceased kidney transplantation in 2007, (CMV donor+/recipient+). Initial immunosuppressive therapy was daclizumab, prednisolone, MMF, and TAC. There were no delayed graft function, acute rejection episodes, and surgical or infectious complications and renal allograft function remained normal.

**Figure 2:**
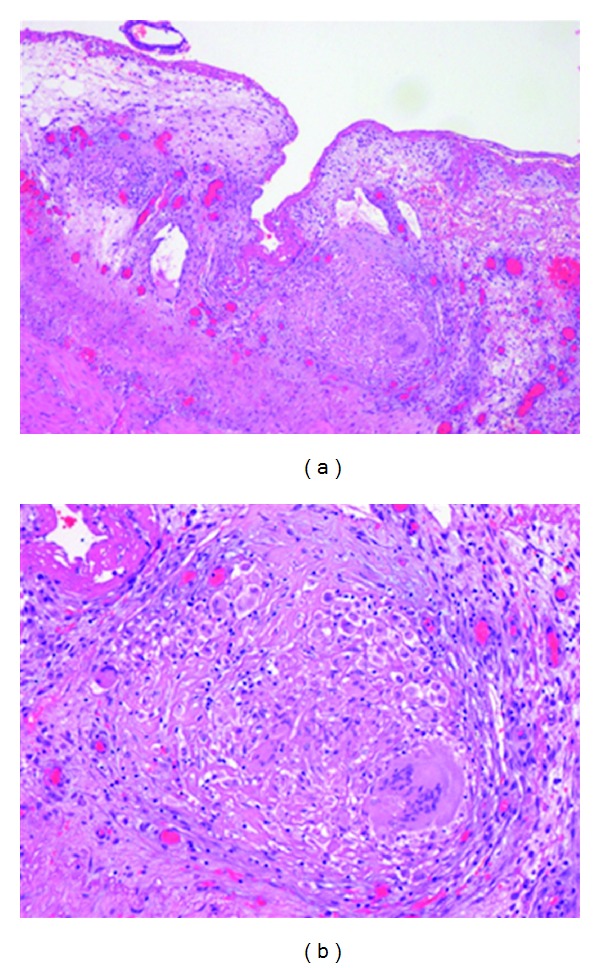
(a) Nonnecrotizing epithelioid granulomas in the ileum (subserosa). (b) Nonnecrotizing epithelioid granulomas in the ileum (subserosa).

Three years after RT, in February 2010, an episode of intestinal obstruction was documented and assumed as secondary to adhesions. Laparotomy was performed and showed few small bowel and colon loops with a whitish appearance and nodular structures of 5 mm in diameter along the ICV. Partial resection of the ileum was done. Histological examination revealed nonnecrotizing epithelioid granulomas in the ileum's subserosa (Figures 2(a) and 2(b)) and a mesenteric nodule showed a necrotizing nongranulomatous inflammation, and Ziehl-Neelsen was negative. After a short period of paucity of abdominal complaints, the patient suffered low-grade fever, pain in the lower abdomen, alternating constipation, and no hemorrhagic diarrhea.

Two months later, in April 2010, she was readmitted with high fever (38.8°C), anorexia, and diffuse abdominal tenderness, especially at the right iliac fossa. Analytic study revealed leukocytosis (12.750/*μ*L), elevated CRP (58 mg/L), without renal graft dysfunction or other abnormalities. Prior pathologic analysis of the intestine was reviewed and PCR analysis was positive for MT. A quadruple therapy consisting of rifampicin, isoniazid, pyrazinamide and ethambutol was started, with favorable evolution. There was a need to increase dose of TAC by 5 times, with no rejection episodes. Six months after anti-TB therapy, small bowel transit and colonoscopy showed no pathological changes. After anti-TB therapy suspension, the patient is asymptomatic, with no signs of reinfection.

## 4. Discussion

 Mycobacterium tuberculosis, with a prevalence of 0.3–1.7% in United States and Western Europe, is a well-known opportunistic agent following RT [9]. As previously described, pulmonary disease is the most frequent, but in RT recipients extra-pulmonar and disseminated disease are common and occur in one-third to half of cases [7].

Gastro-intestinal tuberculosis (GITB) is an infrequent manifestation of TB but a potentially lethal one [9]. The prevalence of GITB in RT patients is about 0.2–0.6% (in developing countries), occurring about 50 times more frequently than in general population [8, 10]. It has a higher frequency during the first post-transplant year (32–57% of all cases) [1, 9] and may be related to higher doses of IS used in initial period and during episodes of acute rejection [9]. However, like in our cases, there are reports of GITB occurring several years after RT. Singh and Paterson [1] attribute the lower IS used in RT, compared to other solid organ transplantation, as a reason for this later presentation. Other authors defend that late presentation may be due to a delay in the diagnosis, as nonspecific symptoms, imaging [9], and difficult differential diagnosis (e.g., with Crohn's disease) [11] may be confounders.

In nontransplant population, the most common symptoms of GITB are abdominal pain, anorexia, fever, and change in bowel habits, reflecting a pattern of inflammatory presentation [12]. However, in RT recipients gastrointestinal bleeding, fever, and abdominal pain are the most frequent complains revealing a predominant ulcerative feature of the disease and reflecting the decreased inflammatory response in immunocompromised patients [12]. Intestinal obstruction, as presented in case 2, is an extremely rare presentation of GITB in RT patients [11], reflecting an indolent evolution leading to late diagnosis.

Radiological findings of GITB include mucosal nodularity or ulceration, particularly of the terminal ileum. CT may demonstrate inflammation, ascites, and lymphadenopathy [13, 14]. These radiographic features are nonspecific and may mimic neoplastic, inflammatory or infectious diseases caused by nontuberculous bacteria, virus, or parasites [1, 13]. As described in our patients, the ileocecal region is the most frequently affected in GITB, with the ileocecal and jejunoileal regions comprising >75% of GITB in the general population [1, 13].

Definitive diagnosis of TB involves the isolation of MT but microbiological confirmation is often hard to accomplish, due to difficulty in obtaining suitable tissue samples, low sensitivity of direct microbiological examination, and prolonged time for cultural results (up to six weeks) [15]. 

In immunosuppressed patients (such as transplant candidates on dialysis or RT recipients), skin anergy is frequent, tuberculin skin testing is often negative [16], and the use of invasive diagnostic techniques, such as bronchoscopy and tissue biopsy, is almost universal. Colonoscopy is less invasive and allows observation of the mucosa and biopsy of suspicious lesions. Colonoscopic findings include ulcers with variable dimensions (from a few millimeters to several centimeters) [17], small diverticula (3–5 mm), sessile polyps, nodules, and mucosal strictures [12, 18].

Endoscopic distinction between GITB and Crohn's disease is extremely difficult and PCR may be useful in differential diagnosis [19]. PCR may be used to identify MT in blood, urine [18], stool cultures [20] and tissue samples. DNA amplification using PCR allows rapid and accurate diagnosis of infections due to organisms that require cellular or complex medium culture, prolonged incubation times or for those in which culture is too insensitive [17]. However, these are expensive techniques that require expertise and may not differentiate between an active infection and a latent or dead MT organism during antibiotic treatment [21].

Several risk factors have been associated with GITB in immunocompromised RT patients [9]. Longer time on HD (due to immunosuppressive effects of ESRD and HD) [22–24], pretransplant diabetes mellitus, and chronic liver disease [6, 25] have been pointed as risk factors for developing post-transplant TB. Opportunistic infections (such as caused by CMV, *Pneumocystis jiroveci,* and *Nocardia*) [6, 26] after RT and the presence [22, 25] and number [23] of acute rejection episodes (with IS augmentation) were also associated with increased risk of TB.

Immunosuppressive therapy, by tapering the function of cell-mediated immunity, may contribute to reactivation of latent infections. The use of CyA during the first posttransplant year [6, 25] and the newer IS agents, such as monoclonal and polyclonal antibodies [27–30], have been associated with TB reactivation. 

Several risk factors were identified in our patients. In both, induction therapy with daclizumab (anti-CD25 monoclonal antibody), immunosuppressive regimen that included calcineurin inhibitors in both patients, and infectious complications (CMV infection in case 1 and hepatitis B in case 2) may have contributed to increased risk of TB. Additionally, in case 2, a long time on dialysis prior to RT (24 years) predisposed her to MT infection.

In immunosuppressed patients, TB is frequent and rapidly disseminates. Anti-TB therapy should be instituted as early as possible, when there is a high degree of clinical suspicion, even in the absence of isolation of MT [31]. The TB treatment proposed for kidney transplant patients is the same initial quadruple therapy used in general population, comprising the administration of isoniazid, rifampicin, ethambutol and pyrazinamide for 2 months, followed by administration of isoniazid and rifampicin for a minimum period of 4 months [1, 31–34]. 

Anti-TB treatment involves risks and toxic hepatitis is one of the major problems [35]. Rifampicin, a first-line antimycobacterial agent, is an inducer of the cytochrome P450 (CYP450) enzyme and increases calcineurin-inhibitor metabolism [32]. Rifampicin association may decrease by 2–5-fold cyclosporine [33] and by 4–10-fold TAC [34] serum concentrations and predispose RT recipients to acute rejection. A careful monitoring and adjustment of calcineurin inhibitors trough concentrations is warranted to avoid rejection and toxicity. Quinolones have been used as a viable alternative to Rifampicin, with the advantage of avoiding increases in the dose of immunosuppressants, and, consequently, increases in the costs of therapy [27].

Although treatable, GITB is potentially lethal [1]. In RT, mortality associated with GITB is extremely high and varies between 20% and 30% [1, 35]. Early diagnosis is fundamental for successful treatment.

In conclusion, the difficulty to make the diagnosis of GITB and the high mortality associated with this infectious disease in RT patients require that rigorous diagnostic investigation should be conducted, in order to allow the early institution of anti-TB therapy. Tuberculosis colitis must always be taken into account when treating RT patients with gastro-intestinal symptoms, especially in the presence of other comorbidities and risk factors.
